# Spectrophotometric Evaluation of the Color Stability of Nanofilled Composites With and Without a Dentin Bonding Agent as a Surface Sealer Following Exposure to Common Beverages

**DOI:** 10.7759/cureus.73591

**Published:** 2024-11-13

**Authors:** Abdulaziz S Alrashidi, Bilal Arjumand, Anas S Al-Mushayqih, Bandar S Alharbi

**Affiliations:** 1 Dentistry, Private Practice, Buraydah, SAU; 2 Department of Conservative Dental Sciences, College of Dentistry, Qassim University, Buraydah, SAU

**Keywords:** chromogenic beverages, color stability, composite sealer, dentin bonding agent, discoloration, nanofilled composite, resin composite, spectrophotometry

## Abstract

Objective: The aim of this study is to evaluate the color stability of nanofilled composites with and without the application of a dentin bonding agent (Single Bond Universal Adhesive) as a surface sealer, following exposure to common beverages.

Methods: Eighty specimen disks of a nanofilled composite (Filtek 350XT) were prepared and divided into two groups: one treated without a composite sealer (Group A) and one with a composite sealer (Single Bond Universal Adhesive) (Group B). Each group was subdivided into four subgroups (subgroups A1, A2, A3, and A4 and subgroups B1, B2, B3, and B4), and each subgroup was immersed in a separate container containing coffee, tea, cola, or distilled water for 15 days. Color changes (ΔE) were measured using a spectrophotometer at baseline and after 15 days. Statistical analysis was performed using one-way analysis of variance (ANOVA) followed by Tukey’s honestly significant difference (HSD) test.

Results: Statistically significant differences in color stability were observed between the sealed and unsealed groups. Specimens without sealant (subgroups A1, A2, and A3) showed greater color changes compared to sealed specimens (subgroups B1, B2, and B3). Coffee caused the greatest color change (ΔE = 30.31 for A1 vs. 12.00 for B1), followed by tea and then cola. Control groups immersed in distilled water showed minimal color change, which was not statistically significant (p > 0.05).

Conclusions: The results suggest that applying Single Bond Universal Adhesive as a composite sealer significantly reduces discoloration of nanofilled composites when immersed in coffee, tea, and cola over a 15-day in vitro period. Further research is needed to explore its long-term efficacy.

## Introduction

The use of composite restorative materials is common in the dental field. They have a wide range of clinical applications. They are used to restore lost tooth structures due to trauma and carious and non-carious lesions. In addititon, they are used for cosmetic procedures such as direct and indirect veneers and diastema closure. They are available in various shades and opacities, which makes them suitable for use in the anterior aesthetic region [1-3]. 

When a clinical decision is made to place a composite restoration in the anterior aesthetic area, both the practitioner and the patient expect the restorative material to have a stable color and to maintain that color over time. A discolored restoration is considered unsuccessful, and noticeable color changes are one of the primary reasons for replacing restorations in the aesthetic area [4,5]. 

Composite restorations are prone to discoloration [4]. Discoloration can manifest in three forms: external, surface or subsurface, and internal. External and surface and subsurface discolorations are caused by plaque, surface stains, and superficial degradation of materials. This is mainly due to dietary choices, such as the consumption of chromogenic beverages (e.g., coffee, tea, and soft drinks), habits like smoking, and poor oral hygiene practices [6,7]. Such factors can lead to the adsorption of chromogens and superficial wear of the material, compromising both its appearance and integrity. On the other hand, internal discoloration often arises from reactions within the composite matrix itself, such as when oxidation occurs in the unreacted components of the resin before they are fully cured. This can cause the color of the restoration to shift to undesirable shades or make it more susceptible to discoloration. Another factor that may contribute to the discoloration of composite materials is composite composition, specifically the type and size of the inorganic filler particles. Larger filler contents may create rougher surfaces that are more prone to staining, whereas smaller fillers can produce smoother surfaces that resist discoloration more effectively [4,8-10]. 

Resin composite restorative materials consist of three main components: the polymerizable resin, the filler content, and the filler-resin interface. Each of these components contributes to the overall properties of the material. Research and developments in each of these components have led to the formulation of different composite restorative materials [11]. Advancements in nanotechnology have resulted in the development of nanocomposites featuring nanofillers sized between 0.1 and 100 nm. It is believed that the size of the nanofiller reduces the voids within the resin matrix, which in turn improves the bond strength and durability of the material. Furthermore, nanofillers enhance the optical properties of the material, resulting in better translucency and aesthetics. Studies have shown that nanofilled composites exhibit superior surface quality, better wear resistance, and improved color stability [12-18].

In the literature, the application of surface sealants on resin composite restorations is often described as a method to reduce susceptibility to discoloration. These sealants have various formulations, and no single formulation is universally accepted. However, they are generally light-cured, low-viscosity resins, either filled or unfilled, containing different types of low-molecular-weight monomers. They are suggested to provide a protective layer over the restoration, fill any surface irregularities, and seal the restoration against water sorption, thereby improving its resistance to staining and discoloration [19-21]. However, when it comes to studies, the results are mixed. Some studies have demonstrated beneficial effects [22-24], while others have shown no effect [25-29], and some concluded that there are detrimental effects [30-33].

It has been suggested that the wetting properties of the monomer used in sealants affect their ability to resist discoloration. Hydrophilic monomers tend to absorb more water, leading to increased discoloration, whereas hydrophobic monomers decrease discoloration as they tend to repel water and reduce discoloration [33]. Single Bond Universal Adhesive (3M ESPE, Neuses, Germany) is notable for its claimed hydrophilic-hydrophobic properties. According to the manufacturer, the water affinity of this dentin bonding agent is hydrophilic before curing and hydrophobic after curing [34]. The hydrophilicity of dentin bonding agents is important for increasing their wettability, which in turn enhances bond strength initially [35]. However, continuous hydrophilicity can negatively affect long-term bond strength [36]. The unique amphiphilic properties of Single Bond Universal Adhesive (3M ESPE, Neuses, Germany) may result in a decreased need for materials and more streamlined clinical steps to achieve a more color-stable restoration. It has a good bond strength [37,38] and a hydrophobic nature after curing, as proposed by the manufacturer [34], which may suggest a potential application as a surface sealant. Bora et al. studied the color stability of nanofilled and two nanohybrid composites with multiple surface sealants containing different types of monomers. They concluded that the nanofilled composite with a surface sealant containing di-gentaerythritol penta-acrylate esters (a hydrophobic monomer) resulted in the least color change [19].

One common way to assess the color stability of composite materials is by utilizing a spectrophotometer to gather and compare measurements in the CIELAB color system. This system consists of three parameters: L*, which denotes luminosity; a*, which indicates red-green saturation; and b*, which represents blue-yellow saturation. The overall color difference (ΔE) is calculated based on the changes in these parameters and expressed as a single value [5,39,40].

Given the current data and understanding, the aim of this study is to evaluate the color stability of nanofilled composite with and without the application of a dentin bonding agent (Single Bond Universal Adhesive) as a surface sealer, following exposure to common beverages. The results of this study could identify an effective composite sealer and help simplify clinical procedures by reducing the number of materials required. This could streamline the restoration process and potentially improve both the efficiency and outcomes of composite restorations in dental practice.

The null hypothesis (H₀) was the application of a dentin bonding agent as a surface sealer does not significantly affect the color stability of nanofilled composites when exposed to common beverages. The alternative hypotheses were (H₁) that the application of a dentin bonding agent as a surface sealer significantly improves the color stability of nanofilled composites when exposed to common beverages and (H₁a) the application of a dentin bonding agent as a surface sealer significantly worsens the color stability of nanofilled composites when exposed to common beverages.

## Materials and methods

Sample preparation

One type of nanofilled composite restorative material (Filtek 350XT Enamel Opacity, Shade: A3. 3M ESPE, Dental Products, Saint Paul, MN, USA) was used. Eighty specimen disks (n = 80) were fabricated, each with a diameter of 7 mm and a thickness of 1 mm. To produce the disks, the composite material was placed into a plastic mold that was hollow on both sides. To produce a smooth surface, a Myler matrix strip was inserted on each side, sandwiching the material. Then, a microscopic slide was placed on each side facing the matrix strip, and the disks were pressed together. The disks were then light-cured using a fully charged light-emitting diode (LED) curing unit based on the manufacturer's instructions for 20 seconds. A new Myler strip was used for each disk. To ensure that the composite syringe remains uncontaminated and is not partially cured by ambient light during the preparation of the sample disks, the tip was covered with its protective cap immediately after placing the required amount inside the mold.

Out of the 80 specimens, 40 of them (n = 40) were treated with an additional step. After light curing, a dentine bonding agent (Single Bond Universal Adhesive, 3M ESPE, Neuses, Germany) was applied as a composite sealer. Two drops of the bonding agent were placed on a cover glass, and a microbrush applicator was used to scrub both sides of the disks for 20 seconds, gently air-blown for five seconds, and light-cured for 10 seconds, based on the manufacturer's instructions. A new cover glass and microbrush applicator were used for each disk, and a vial cap was used between each application to ensure no contamination. All the specimens were left for 48 hours in sealed containers to ensure complete polymerization before any immersion.

The group of 40 disks that were not treated with the composite sealer was designated as Group A, while the other 40 disks that received the composite sealer treatment were designated as Group B.

Solution preparation and storage

To further categorize the groups, Group A was subdivided into four subgroups named A1, A2, A3, and A4, while Group B was also subdivided into four subgroups named B1, B2, B3, and B4. Each subgroup contained 10 specimens (n = 10).

Four different solutions were prepared: coffee (Nescafe Red Mug Instant Coffee, Nestle UAE LLC, Saudi Arabia) by using 5 grams of ground coffee in 250 ml of boiled water and stirred, tea (Rabea, Ahmad Mohamed Saleh Baeshen and Co. Tea industried, Jeddah, Saudi Arabia) by using 1 tea bag in 250 ml of boiled water, cola (Coca-Cola can 325 ml, Coca Cola Bottling Company of Saudi Arabia, SA Grocery, HS1DI, Saudi Arabia), and distilled water (Brady, Mama Sharouq for Industrial Factory, Saudi Arabia) as a control solution. 

All solutions were left to cool to room temperature. Then, the subgroups were immersed in these solutions using 100 ml plastic containers. Specifically, subgroups A1 and B1 were each immersed in separate containers of coffee, subgroups A2 and B2 were each immersed in separate containers of tea, subgroups A3 and B3 were each immersed in separate containers of cola, and subgroups A4 and B4 were each immersed in separate containers of distilled water, which served as the control subgroups. These containers were sealed and then left at room temperature for 15 days. After 15 days, the specimens were rinsed and dried with tissue papers.

Color value determination, data storage, and statistical analysis

A spectrophotometer (VITA Easyshade Advance 4.0) was used to measure the CIE Lab* color space values of each specimen. For standardization of the measurements, each specimen’s color was measured against a grey card (WhiBal G7 White Balance Pocket Card) after the device was calibrated. The color values were measured at the baseline before the specimen immersion and after 15 days. The color change (ΔE) was calculated for each specimen using the formula ΔE=[(ΔL*)^2+(Δa*)^2+(Δb*)^2]^0.5]. All color values were recorded in a Microsoft Excel sheet (Microsoft Corp., USA).

The data were analyzed using IBM SPSS Statistics for Windows, version 20.0 (released 2011, IBM Corp., Armonk, NY) with one-way ANOVA followed by Tukey’s HDS test. A one-way analysis of variance (ANOVA) was used as the statistical method because this study involves comparing the means of multiple subgroups (A1, A2, A3, A4, B1, B2, B3, and B4) to determine if there are statistically significant differences in color change (ΔE) between them. ANOVA tests the overall effect of the independent variable (solution type and/or treatment) on the dependent variable (color change). Based on the results of previous studies using similar independent variables, a statistically significant difference was expected. Therefore, Tukey’s honestly significant difference (HSD) test was used for pairwise comparisons to identify exactly which pairs of groups have significant differences in their means.

## Results

Between-group comparison

**Figure 1:**
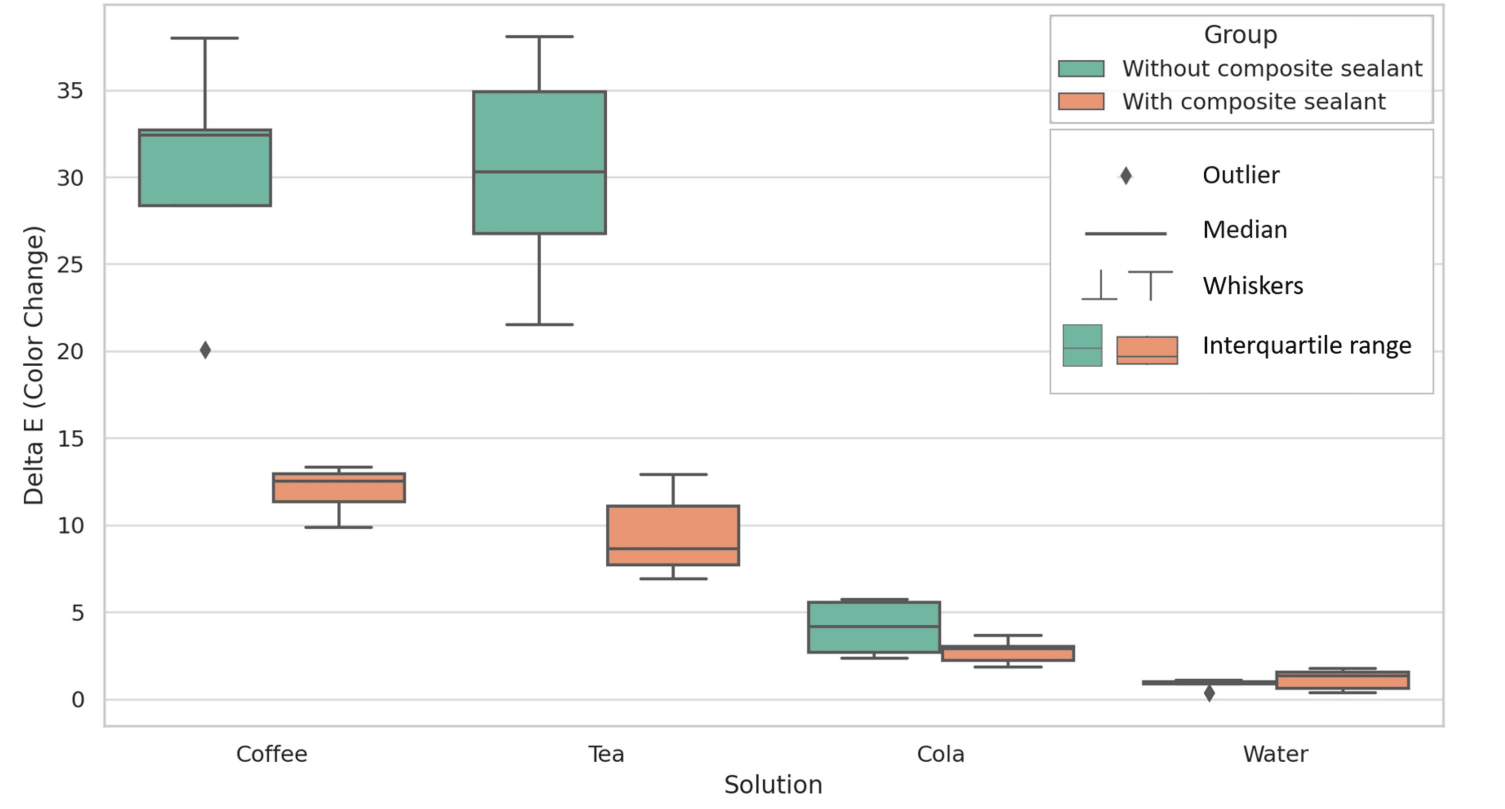
Boxplot comparison of the distribution of color change (ΔE) values among the different groups. Each box in the boxplot represents the interquartile range (IQR), which contains the middle 50% of the data. The horizontal line inside the box marks the median, which divides the data into two equal halves. For each subgroup, this line indicates the central tendency of the ΔE values. The whiskers extend from the edges of the box to show the spread of the data beyond the IQR. Specifically, the whiskers represent the smallest and largest values that fall within 1.5 times the IQR from the quartiles. Data points beyond this range are considered outliers. An outlier is marked by an individual point outside the whiskers, indicating a value that deviates significantly from the rest of the data. The boxplot reveals a wider spread of ΔE values for the unsealed subgroups (A1, A2, A3), especially in the coffee and tea subgroups, where the whiskers extend farther, showing a higher level of variability in color changes. In contrast, the sealed subgroups (B1, B2, B3) exhibit a narrower distribution of ΔE values, with the whiskers being shorter, indicating more consistent color changes across these groups. In the control subgroups (A4, B4), the boxplot shows minimal color change, as evidenced by the tight clustering of ΔE values, where the box and whiskers are compact, suggesting that most of the data points are close to the median and the color change is small.

**Figure 2:**
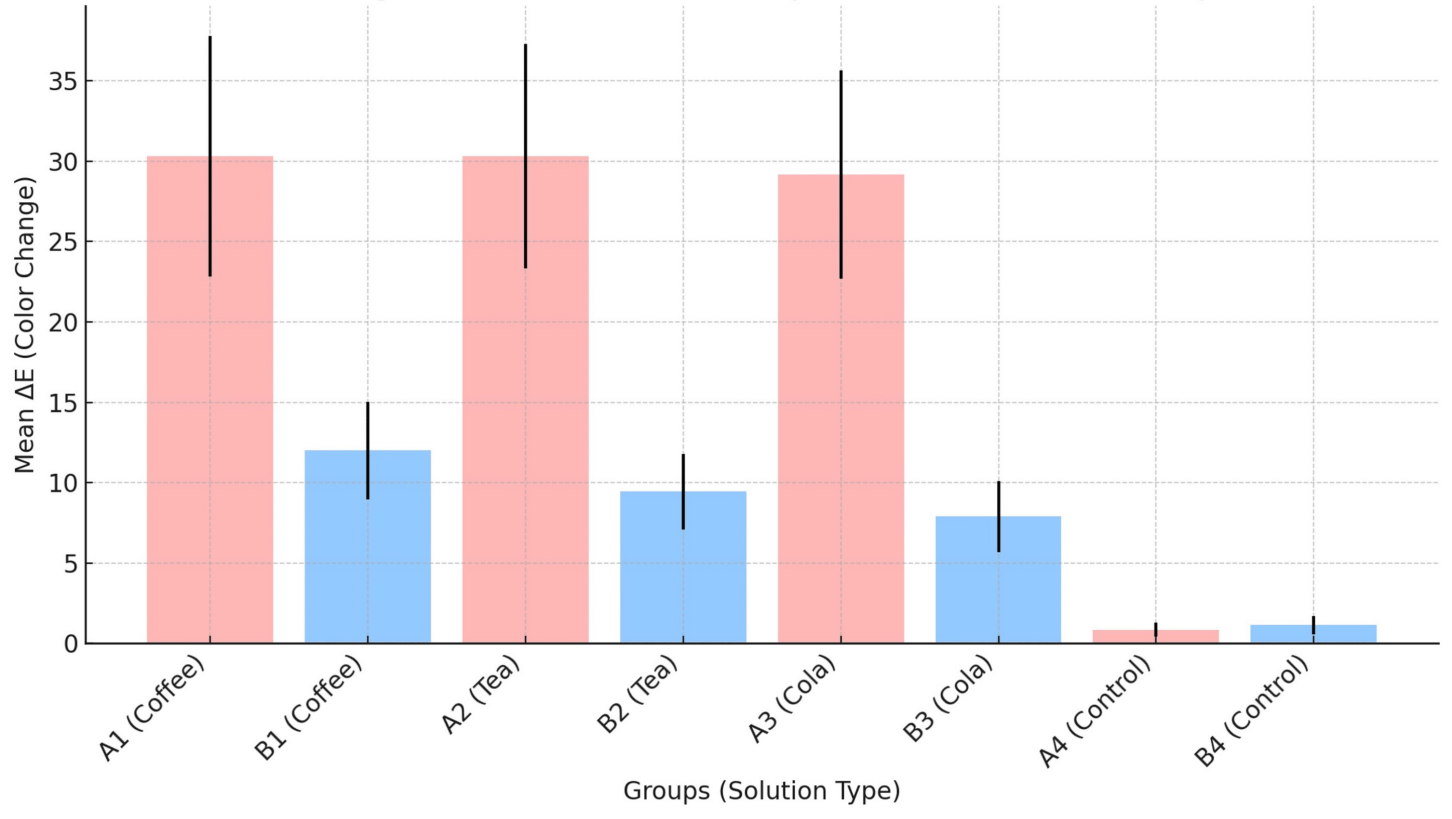
Bar graph illustrating the mean color change (ΔE) values across the different groups The graph compares groups with and without composite sealant after immersion in coffee, tea, cola, and distilled water (control). The unsealed subgroups (A1, A2, A3, A4) are shown in red, while the sealed subgroups (B1, B2, B3, B4) are displayed in blue. The error bars represent the standard deviation, providing a clear visualization of the variability within each subgroup. The graph demonstrates that the subgroups without sealant (A1, A2, A3) experienced significantly greater color changes, particularly after immersion in coffee, tea, and cola, compared to the sealed subgroups (B1, B2, B3). By contrast, the control subgroups (A4, B4) immersed in distilled water showed minimal color change, with no significant difference between sealed (B4) and unsealed subgroups (A4).

A one-way ANOVA revealed a significant difference in color change (ΔE) values between the subgroups (F = 138.450, p < 0.001). Post-hoc comparisons using the Tukey HSD test showed significant differences between test subgroups, with 95% confidence intervals for the mean differences that did not include zero. Figure 1 provides a boxplot comparison of the distribution of color change (ΔE) values among the different subgroups. Figure 2 is a bar graph that illustrates the mean color change (ΔE) values across the different subgroups.

Tukey’s HSD

Tukey’s HSD post-hoc analysis confirmed that the subgroups immersed in coffee, tea, and cola without sealant (groups A1, A2, A3) exhibited significantly higher ΔE values than their sealed counterparts (groups B1, B2, B3) (p < 0.001 for all). Specifically, specimens immersed in coffee exhibited the largest color changes, with subgroup A1 (without sealant) showing a mean ΔE of 30.31 ± 7.48, while subgroup B1 (with sealant) had a mean ΔE of 12.00 ± 3.05. The difference between these two subgroups was statistically significant (p < 0.001). 

A similar pattern was observed in tea immersion, where subgroup A2 (without sealant) had a mean ΔE of 30.30 ± 6.98, compared to subgroup B2 (with sealant), which had a mean ΔE of 9.45 ± 2.34. This difference was also statistically significant (p < 0.001). Cola immersion resulted in subgroup A3 (without sealant) having a mean ΔE of 29.16 ± 6.48, while subgroup B3 (with sealant) exhibited a mean ΔE of 7.90 ± 2.21, with a statistically significant difference (p < 0.001). 

By contrast, the control subgroups immersed in distilled water showed the smallest color changes, with subgroup A4 (without sealant) having a mean ΔE of 0.85 ± 0.43, and group B4 (with sealant) showing a mean ΔE of 1.14 ± 0.59; however, there was no statistically significant difference between these two subgroups (p > 0.05).

## Discussion

The aim of this study was to evaluate the color stability of nanofilled composite with and without the application of a dentin bonding agent (Single Bond Universal Adhesive) as a potential surface sealer, following exposure to common beverages. The results demonstrate that the unsealed subgroups (A1, A2, A3) exhibited statistically significant color change (ΔE). As seen in Figure 2, the sealed subgroups (B1, B2, B3) have lower mean color change (ΔE), indicating less variation and better overall color stability compared to their unsealed counterparts (A1, A2, A3). These findings suggest that the application of the Single Bond Universal Adhesive as a composite sealer provided protection against discoloration in an in vitro setting. Thus, the null hypothesis (H₀) and the second alternative hypothesis (H₁a) were rejected. The first alternative hypothesis (H₁) was accepted.

The significant differences in color change (ΔE) after exposure to coffee, tea, and cola align with previous research. Poggio et al. highlighted coffee and tea as chromogenic substances contributing to noticeable discoloration in resin-based materials [4]. Similarly, Assaf et al. found that beverages like coffee and cola induce significant staining in resin composites [8]. Coffee exhibited the most severe discoloration (ΔE = 30.31 in the unsealed subgroup versus 12.00 in the sealed subgroup), which might be due to its acidic nature and the presence of chromogenic compounds such as tannins, making coffee potent in staining resin-based materials [41].

Regarding color change (ΔE), the perceptibility threshold (PT) is defined as the smallest color difference that is detected by the human eye, whereas the acceptability threshold (AT) is defined as the level of color difference that is considered acceptable in a given application. In the dental field, there are no universally accepted PT and AT limits. However, for PT, it is reported to be ΔE ≥ 2.6 [42], ≥ 3.3 [43], and ≥ 3.7 [44], while AT limits are ΔE < 5.5 [42] and < 6.8 [44]. A recent comprehensive review reported it to be ≥ 1.2 for PT and < 2.7 for AT [45]. In our study, all subgroups, except the control subgroups, exhibited ΔE < 6.8, which indicates a perceptual and unacceptable color difference. However, this is a common finding in studies that use immersion in similar chromogenic beverages for 15 days or even less [4,46,47]. This marked color change can be mainly attributed to the increased contact time between the specimens and the discolorant. Immersion in a chromogenic beverage greatly intensifies the effects of their discoloration potential. It has been reported that the effects of discoloration from immersion in coffee for 15 days are equivalent to the intraoral effects of consuming coffee for over one year [48].

As for the effects of the composite sealer, Bora et al. studied the effect of different types of composite sealers on the color stability of two nanohybrids (Clearfil Majesty ES-2, Kuraray, Osaka, Japan, and Charisma Topaz, Kulzer, Hanau, Germany) and one nanofilled (Filtek Ultimate, 3M, MN, USA) composite when immersed in coffee. They included in their study three types of sealers: permaseal (Ultradent Products, UT, USA), which is an unfilled sealer with a composition of Bis-GMA 60%, TEGDMA 40%, 1-dimethylaminoethyl methacrylate < 3%; Biscover LV (Bisco, IL, USA), which is an unfilled sealer with a composition of dygentaerythritol penta-acrylate esters and ethanol; and Optiglaze Color Clear (GC Corp., Tokyo, Japan), which is a nanofilled sealer with a composition of methyl methacrylate (30-40%), silica filler (10%), and multifunctional acrylate (50-60%). They reported that Biscover LV resulted in the least amount of color change when combined with Filtek Ultimate nanofilled composite, followed by Optiglaze Color Clear when combined with Charisma Topaz nanohybrid composite, while the highest color change was observed in Permaseal combined with Clearfil Majesty ES-2 nanohybrid composite [19]. Gunce et al. reported that after weeks of thermocycled coffee immersion, Biscover LV and Adper Single Bond 2 as a surface sealer (3M ESPE, St. Paul, MN, USA) resulted in the lowest color change compared to other groups [49].

The color stability of resin-based materials is influenced generally by the following factors: the various types of monomers in their matrix, water affinity, and the degree of conversion [50]. For example, Bis-GMA (bisphenol A-glycidyl methacrylate), a hydrophilic monomer present in many composite materials and sealers, is more prone to discoloration compared to UDMA, which is less hydrophilic [23,51]. By contrast, dipentaerythritol pentaacrylate is less prone to discoloration, although the mechanism was not explained. It might be said that it is less prone to discoloration due to its dipentaerythritol structure, as it primarily consists of carbon-carbon (C-C) and carbon-hydrogen (C-H) bonds, which are nonpolar molecules, making it hydrophobic [19, 52]. The degree of conversion is the ratio of reacted monomers to unreacted monomers, evaluated by comparing the number of C=C double bonds remaining in a polymerized sample to the total number of C=C bonds in the original nonpolymerized sample, and is expressed as a percentage [53]. A low degree of conversion is known to increase discoloration in resin-based materials due to the unreacted monomers, which are more prone to discoloration and may cause internal discoloration [46,54,55]. Regarding Single Bond Universal Adhesive (3M ESPE, Neuses, Germany), it is claimed that it has a high degree of conversion, ranging from 83% ± 4% to 85% ± 5% depending on the substrate. It is reported to be 77.2 ± 16.5 and 76.3 ± 13.7 [56], with a hydrophobic nature after curing [34]. The exact process and mechanism are not disclosed by the manufacturer, nor is the type of dimethacrylate monomer specified. However, it might be due to the high degree of conversion, indicating that a greater proportion of the monomers are transformed into a polymer network during curing, resulting in fewer unreacted monomers that could contribute to discoloration over time. In addition, the formulation may include hydrophobic aliphatic dimethacrylates, which may increase the cross-linking density between the polymers, creating a tightly structured barrier that further resists color changes by limiting the diffusion of staining agents [57]. However, the presence of HEMA (2-hydroxyethyl methacrylate) in its composition is attributed to increased hydrophilicity and possible hydrolytic degradation [58].

In this study, no polishing was attempted for two reasons. First, for standardization purposes, it is difficult to standardize the pressure, angle, and speed of the strokes from the polishing devices. Second, studies have reported that the smoothest surface can be achieved by pressing the composite material against a Mylar strip, which was adopted in this study [59-62].

Despite the findings of this study, several limitations should be acknowledged. First and foremost is the uncertainty regarding the long-term effects of Single Bond Universal Adhesive as a composite sealer, due to the presence of 2-hydroxyethyl methacrylate in its composition, which is reported to be susceptible to hydrolytic degradation [63]. This might explain the slight color change in the sealed control group as seen in Figure 2, although not statistically significant. Second, the specimens were handled by holding them at the periphery to prevent any damage to the sealer at the sides. For this reason, no sealer was applied at the periphery. However, as the periphery was not sealed, this might have introduced some variation, potentially due to water sorption. In addition, only one type of composite restorative material was used, as different types of composite restorative materials may exhibit different results. No other types of sealers were used, which might have provided better insight into the efficacy of Single Bond Universal Adhesive as a composite sealer. Furthermore, the correlation of in vitro studies to clinical settings should always be approached with caution. The staining protocol in this study might not accurately reflect the oral environment, and the in vitro design does not take into account the complex oral environment, including the effects of saliva flow, mechanical abrasion, mastication, dietary habits, and oral hygiene practices. Moreover, it is important to note that, to the best of our knowledge, the use of a dentin bonding agent as a surface sealer in composite restorations does not reflect standard clinical practice. While it has been used in previous in vitro studies as a surface sealer [49], its use remains experimental and not yet established in clinical practice.

Further studies are necessary to evaluate the long-term effects of Single Bond Universal Adhesive as a composite sealer, to compare it with different types of composite restorative materials and sealers, and to assess its performance in both simulated in vitro studies and clinical settings, in order to validate the findings of this study.

## Conclusions

Within the limitations of this study, the results suggest that applying Single Bond Universal Adhesive as a composite sealer significantly reduces discoloration of nanofilled composites when immersed in coffee, tea, and cola over a 15-day in vitro period. The sealed composites consistently exhibited lower color changes compared to the unsealed specimens. These findings support the potential effectiveness of Single Bond Universal Adhesive in enhancing the color stability of nanofilled composites against common staining beverages. However, further research is needed to assess its long-term efficacy and to compare these findings with those of other sealers and composite materials.
